# An educational intervention impact on the quality of nursing records

**DOI:** 10.1590/1518-8345.1986.2938

**Published:** 2017-10-30

**Authors:** Graciele Fernanda da Costa Linch, Ana Amélia Antunes Lima, Emiliane Nogueira de Souza, Tais Maria Nauderer, Adriana Aparecida Paz, Cíntia da Costa

**Affiliations:** 1 PhD, Adjunct Professor, Departamento de Enfermagem, Universidade Federal de Ciências da Saúde de Porto Alegre, Porto Alegre, RS, Brazil.; 2 Master’s student, Departamento de Enfermagem, Universidade Federal de Ciências da Saúde de Porto Alegre, Porto Alegre, RS, Brazil. RN, Irmandade Santa Casa de Misericórdia de Porto Alegre, Porto Alegre, RS, Brazil.

**Keywords:** Nursing Process, Nursing Records, Education Nursing

## Abstract

**Objective:**

to evaluate the impact of an educational intervention on the quality of nursing records.

**Method:**

quasi-experimental study with before-and-after design conducted in a hospital. All the nurses in the cardiac intensive care unit of the hospital received the intervention, which consisted of weekly meetings during five months. To collect data, the instrument Quality of Diagnoses, Interventions and Outcomes was applied to the patients’ charts in two moments: baseline and after intervention.

**Results:**

the educational intervention had an impact on the quality of the records, since most of the items presented a significant increase in their mean values after the intervention, despite the low values in the two moments.

**Conclusion:**

the educational intervention proved to be effective at improving the quality of nursing records and a lack of quality was identified in the evaluated records, revealed by the low mean values and by the weakness of some questions presented in the items, which did not present a significant increase. Therefore, educational actions focused on real clinical cases may have positive implications for nursing practice.

## Introduction

Nursing records are part of the nursing care and can benefit the patient, the professional, the institution and the health system. Detailed records help following the progress of the patient’s clinical condition, contributing to the continuity of care and the planned treatment^1^, providing information to evaluate the care delivered and providing data to develop health indicators.

Discussions about the relevance of nursing records and the nursing process came into greater prominence in the 1950’s. However, the importance of nursing records was expressed by Florence Nightingale in the nineteenth century, when she described, on her notes, a formal collection process to assess people’s health history^2^.

A considerable growth in the computerization of clinical documents took place in the 1990’s. However, this is a process gradually developed over time and it is in different stages of implementation, considering that it requires heavy investment in infrastructure, incurs high costs of implementation and technical maintenance and also demands continuous training of its users^3^.

For the computerization of nursing records, it is fundamental to define which standardized language systems (taxonomies) will be used, since they are the guarantee that the nursing process is visible in electronic health records^3^. In addition to favoring the use of structured and standardized methodologies of data, the use of systematized language allows the interoperability between computerized systems, among other factors.

Taxonomies started being used as a way to standardize nursing language in the 1960s. Since then, several terminologies have been used, mostly in order to improve the quality of the care provided, which is directly related to the way the diagnosis, intervention and outcome are registered. The nursing records are continuously improved with the use of technologies and different communication techniques^4^.

Some researchers identify different educational actions focused on improving the quality of nursing records^5^ ^-^ ^6^. Therefore, a continuous development of research and interventions addressing nursing records is important in order to promote its continuous improvement and further improve the scientific bases associated to practice. Thus, the present study was designed to assess the impact before and after an educational intervention on the quality of nursing records.

## Method

Quasi-experimental study with before-and-after design, conducted in a philanthropic hospital complex in the South Region of Brazil, with beds available for the Brazilian Unified Health System (SUS). The study was carried out from the first semester of 2013 to the last semester of 2015.

**Figure 1:**
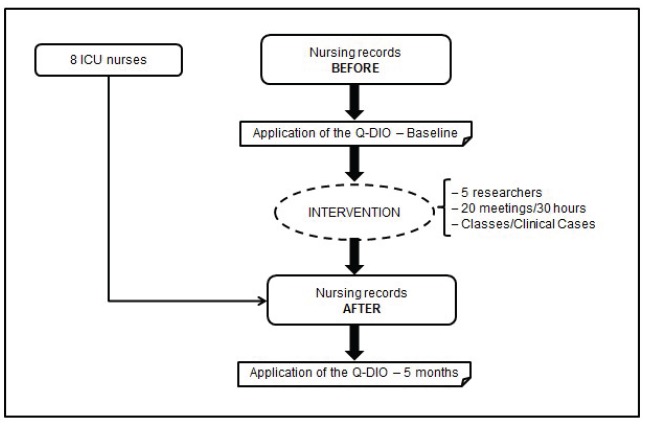
Study design. Porto Alegre, RS, Brazil, 2015

Figure 1 shows the study design, with the description and details of each part of the method executed.

Q-Dio: Quality of Diagnoses, Interventions and Outcomes

The nursing records registered by a group of eight nurses from a cardiac intensive care unit were included in this study. The group was composed of all the nurses who worked in the unit. The nurses were invited to participate in the study and the consent and participation of 100% of the group was obtained. No socio-demographic characteristics were investigated as it was not relevant to the present approach to compare nurses or even to divide them into groups.

The nursing records included were not computerized, they did not use standardized language and they were chosen for convenience, that is, all the nursing records registered by the nurses, in agreement with the collection instrument and available during the collection period were included. The nursing records contained information about hospitalized patients who stayed for at least 48 hours in the unit and had their medical record, history, evolutions and nursing prescriptions recorded during a minimum period of four days. The evaluation started in the first record (admission or first evolution). This period is determined according to the recommendations of the Quality of Diagnosis, Interventions and Outcomes (Q-DIO)^7^ ^-^ ^8^. There was no collection of patient information, such as age or diagnosis, and there were no interviews, since those were not the focus of the study or part of the instrument items. No exclusion criteria were adopted in this study.

The nursing records selected for convenience were assessed by applying the Q-DIO instrument in two moments: baseline (data collection 1), applied before the intervention and at the second data collection, five months after the intervention.

The purpose of the Q-DIO Brazilian version is to evaluate the quality of the documentation of nursing diagnoses, interventions and results. It can be used for electronic or paper records and for nursing records with or without standardized language. This instrument was developed and validated in 2007 and published in 2009 by researchers from Switzerland, the Netherlands and the United States of America^7^. The Q-DIO version for use in Brazil has been published recently^8^. The instrument is a Likert scale composed of 29 items divided in four concepts: nursing diagnoses as process (11 items), nursing diagnoses as product (eight items), nursing interventions (three items) and nursing outcomes (seven items). The scores range from 0 to 2 for all items, 0 being undocumented, 1 partially documented and 2 fully documented^7^ ^-^ ^8^.

## Intervention

The intervention consisted of weekly meetings lasting one and a half hours, during five months, with a total of 30 hours in 20 meetings.

The sessions (interventions) were conducted by a group of five nurses who had a PhD in nursing, were adjunct professors in the nursing department and researchers in the subject under study. These nurses had no working relationship with the hospital, only with the university.

The first two sessions started with a theoretical approach, focusing on clinical evaluation and elaboration of diagnoses, interventions and description of results, using the taxonomies NANDA-I, Nursing Outcomes Classification (NOC) and Nursing Interventions Classification (NIC). At that moment, the group of nurses received theoretical-practical classes, with lectures on the content and discussions about the taxonomies with explanations and use of books, addressing the structure of each of the taxonomies and discussing fictional clinical cases.

The following sessions were conducted based on discussions of actual clinical cases of hospitalized patients in the unit under study, which were presented by nursing assistants and debated with the researchers. The methodology used in the case presentation meetings included strategies for developing critical thinking skills for nursing diagnoses (or problems) and questioning ability to determine signs and symptoms in the cases. The sessions also presented suggestions for effective interventions for the possible etiologies, aiming at achieving better results and, consequently, a better quality of the records and the care offered.

## Data analysis

The data were organized and analyzed using the program Statistical Package for the Social Sciences (SPSS). Continuous variables were described based on mean and standard deviation. The categorical variables were described with absolute and relative frequencies.

The pre and post-intervention periods were compared by the Student’s t-test. The choice for this test considered the evaluation of the data from the normality test. The results were considered statistically significant if p <0.05, with a 95% confidence interval.

## Ethical Considerations

The research was conducted in accordance with the Directives and Norms Regulating Research Involving Human Beings (CNS Resolution No. 466/12). The project was approved by the Research Ethics Committee of the Federal Medical Sciences Foundation of Porto Alegre (CEP-UFCSPA) under protocol (CAAE) number: 20292113.9.0000.5345. Two terms were used: Data Usage Agreement Form (DUAF), which establishes a commitment to use and preserve the material and the Consent Form (CF), which establishes the participation of the study subjects.

## Results

Records of 30 patients were evaluated before and after the intervention. The results presented in Tables 1 to 4 show the mean values and Standard Deviation (SD) for all items, including p value, on the moments pre- and post-intervention.

**Table 1:** Mean and standard deviation of the items in the concept Nursing Diagnoses as Process. Porto Alegre, RS, Brazil, 2015

**Items**	**Before** M*(sd ^**†**^ **)**	**After** M*(sd ^**†**^ **)**	**p** ^**‡**^
**1 - Actual situation, leading to the hospitalization**	**1.53 (0.57)**	**1.53 (0.57)**	**0.001**
**2 - Anxiety and worries related to hospitalization, expectations and desires about hospitalization**	**0.10 (0.30)**	**0.10 (0.30)**	**<0.001**
**3 - Social situation and living environment/ circumstances**	**0.03 (0.18)**	**0.03 (0.18)**	**<0.001**
**4 - Coping in the actual situation/with the illness**	**0.07 (0.25)**	**0.07 (0.25)**	**<0.001**
**5 - Beliefs and attitudes about life (related to the hospitalization)**	**0.00 (0.00)**	**0.00 (0.00)**	**<0.001**
**6 - Information of the patient and relatives/significant others about the situation**	**0.07 (0.25)**	**0.07 (0.25)**	**0.038**
**7 - Intimacy, being female/male**	**-**	**-**	**-**
**8 - Hobbies, leisure activities**	**0.00 (0.00)**	**0.03 (0.18)**	**0.321**
**9 - Significant others (contact people)**	**0.00 (0.00)**	**0.23 (0.62)**	**0.46**
**10 - Daily living activities**	**0.00 (0.00)**	**0.60 (0.49)**	**<0.001**
**11 - Relevant nursing priorities according to the assessment**	**1.30 (0.53)**	**1.30 (0.53)**	**<0.001**

*mean; †standard deviation; ‡p value

**Table 2:** Mean and standard deviation of the items in the concept Nursing Diagnoses as Product. Porto Alegre, RS, Brazil, 2015

**Items**	**Before** M*(sd ^**†**^ **)**	**After** M*(sd ^**†**^ **)**	**p** ^**‡**^
**12 - Nursing problem/nursing diagnosis label is formulated**	**0.80 (0.40)**	**0.97 (0.18)**	**0.045**
**13 - Nursing diagnosis label is formulated according to NANDA** ^**§**^ **and numbered**	**0.37 (0.49)**	**0.97 (0.18)**	**<0.001**
**14 - The etiology is documented**	**0.33 (0.47)**	**0.93 (0.25)**	**<0.001**
**15 - The etiology is correct, related/corresponding to the nursing diagnosis**	**0.50 (0.77)**	**1.87 (0.43)**	**<0.001**
**16 - Signs and symptoms are formulated**	**1.00 (0.00)**	**1.00 (0.26)**	**1.000**
**17 - Signs and symptoms are correctly related to the nursing diagnosis**	**0.80 (0.76)**	**1.87 (0.43)**	**<0.001**
**18 - The nursing goal relates/corresponds to the nursing diagnosis**	**0.07 (0.25)**	**0.23 (0.56)**	**0.148**
**19 - The nursing goal is achievable through nursing interventions**	**0.07 (0.25)**	**0.20 (0.48)**	**0.187**

*mean;†standard deviation; ‡p value; §NANDA - North American Nursing Diagnosis Association

**Table 3:** Mean and standard deviation of the items in the concept Nursing Interventions. Porto Alegre, RS, Brazil, 2015

**Items**	**Before** M*(sd ^**†**^ **)**	**After** M*(sd ^**†**^ **)**	**p** ^**‡**^
**20 - Concrete, clearly named nursing interventions according to NIC** ^**§**^ **are planned**	**0.83 (0.37)**	**0.97 (0.18)**	**0.088**
**21 - The nursing interventions affect the etiology of the nursing diagnosis**	**0.90 (0.75)**	**1.83 (0.46)**	**<0.001**
**22 - Nursing interventions carried out, are documented**	**0.43 (0.50)**	**0.97 (0.18)**	**<0.001**

*mean; †standard deviation; ‡p value; §NIC - Nursing Interventions Classification

**Table 4:** Mean and standard deviation of the items in the concept Nursing Outcomes. Porto Alegre, RS, Brazil, 2015

**Items**	**Before** M*(sd ^**†**^ **)**	**After** M*(sd ^**†**^ **)**	**p** ^**‡**^
**23 - Acute, changing diagnoses are assessed daily or from shift to shift**	**1.57 (0.50)**	**1.87 (0.34)**	**0.009**
**24 - The nursing diagnosis is reformulated**	**0.83 (0.53)**	**1.93 (0.25)**	**<0.001**
**25 - The nursing outcome is documented**	**0.03 (0.18)**	**0.97 (0.41)**	**<0.001**
**26 - The nursing outcome is observably/measurably documented according to NOC** ^**§**^	**0.00 (0.00)**	**0.90 (0.30)**	**<0.001**
**27 - The nursing outcome shows improvement**	**0.03 (0.18)**	**1.23 (0.62)**	**<0.001**
**28 - There is a relationship between outcomes and nursing interventions**	**0.07 (0.36)**	**1.63 (0.71)**	**<0.001**
**29 - Nursing outcomes and nursing diagnoses are internally related**	**0.07 (0.36)**	**1.63 (0.71)**	**<0.001**

*mean; †standard deviation; ‡p value; §NOC - Nursing Outcomes Classification

Table 1 shows the values of items included in the concept Nursing Diagnoses as Process. It should be noted that, for item 7, there was no result in the calculation because the value remained zero in the two moments. In the remaining items, except 8 and 9, there was a statistically significant increase.

Table 2 presents the values of the items included in the concept Nursing Diagnoses as Product. It should be noted that for items 12, 16, 18 and 19 there was no statistically significant increase.

Table 3 shows the values of the items included in the concept Nursing Interventions. It should be noted that only item 20 presented no statistically significant increase.

All the items from the concept Nursing Outcomes displayed in Table 4 presented a statistically significant increase, except for item 23.

## Discussion

The results demonstrated the effectiveness of the intervention, the lack of quality of the records assessed, revealed by the low mean values, ​​and also the weakness of some questions presented in the items of the Q-DIO. This is indicated by the assessment of the 29 items, most of which showed a significant increase in the mean values after the intervention.

Studies have demonstrated that education-focused interventions addressing the nursing process can improve the quality of nursing records^5^ ^-^ ^6^. A quasi-experimental study conducted in a developing country evaluated 40 nurses using a five-day workshop (with concepts for documentation and use of standardized language). In this study, our researchers identified that the combination of education on the use of nursing diagnoses, standardized nursing languages ​​and standardized nursing care plans can improve the documentation of care^6^. Another study, carried out in Switzerland, assessed the effect of Guided Clinical Reasoning as a method to improve the quality of records with the implementation of electronic documentation^5^. In both studies, the Q-DIO instrument was used for evaluation before and after it^5^ ^-^ ^6^.

In the present study, Table 1 presents the items included in the concept Nursing Diagnosis as Process. These items assess the information provided by the patient or relative during the initial interview or anamnesis performed by the nurse, usually guided by a script or clinical history.

The nurses participating in the educational activity, now aware of the improvement of the nursing quality records necessity, received material in a virtual learning environment, including classes and other learning materials to guide the different stages of the nursing process. However, the evaluation of the records registered by these nurses showed that, among the 11 items of the first phase of the Q-DIO, items 7, 8 and 9 presented low averages, indicating poor quality of the registry, either because it is incomplete or because it is partially complete. Item 7 assesses information provided by the patients about their sexual life, and the absence of this piece of information, in both analyzed moments, serving as an alert of the importance of completing the nursing record, since this document contributes to the analysis of the nursing diagnosis hypotheses.

The information regarding hobbies and leisure activities is assessed in item 8, which presents a small variation before and after the intervention. Considering that the patients, whose nursing records were analyzed were hospitalized in a cardiac intensive care unit, information on sexual life, hobbies and leisure are potentially relevant, as patients will need post-discharge instructions for their rehabilitation. From this perspective, the collection of nursing record data will contribute to the processes of decision making and nursing care plans^2^.

Item 9 presents a question considered as fundamental in care: contact information of relatives or significant others. However, the nurses in the present study have not been recording this piece of information. In general, nurses have used the nursing record partially, prioritizing the collection of data regarding some human needs to the detriment of others, with emphasis on biological aspects, and even failing to complete patient and professional identification data. This contributes to the fragmentation of the care provided and hinders the delivery of a more individualized care^9^.

The analysis of the concept Diagnosis as Product found no significant increase in the items 12, 16, 18 and 19. Items 12 and 16 can be discussed under the same perspective, since both address issues fundamental for nursing care and documentation: the description of the problem and the signs/symptoms. However, these items would only receive maximum scores if their records were in accordance with NANDA-I^8^.

A systematic review with the main objective of evaluating the studies and evidences produced according to the five taxonomies validated by the American Nursing Association (ANA) demonstrated that the NANDA-I taxonomy predominates in studies developed worldwide, as well as in studies with higher evidence level^4^. Another study compared the quality of nursing records that used NANDA-I and International Classification for Nursing Practice (ICNP) using the Q-DIO instrument. The study identified that the hospital that used NANDA-I in the records presented a better quality nursing documentation than the other hospital, which used ICNP^10^.

Items 18 and 19 are fundamental, since they address the issue of nursing goals. In the present study, these items did not show significant improvement and also presented values ​​very close to zero, meaning there were no records of nursing goals. Thus, in addition to the fact that nurses did not register those topics, they also failed to describe specific nursing goals for the problems or even achievable objectives through the registered intervention. The nurse will always have a goal already set by the work process when facing a nursing problem. However, the nurses rarely document the goal of the interventions (daily activities). A recent study was developed to assess the records of patients admitted in the medical clinic of a Brazilian teaching hospital. It revealed that, despite the fact the records were in compliance with the norms of the Regional Nursing Council, there were significant flaws related to medical history, physical examination, absence of date or time, blank spaces, spelling errors and non-standard abbreviations^11^. Therefore, it is worth emphasizing that investments in permanent and continuing education are necessary not only to adjust the work process through the systematization of nursing care, but also to find the factors or conditions that represent difficulties or solutions for the production of adequate nursing records. 

A Norwegian study pointed out some weaknesses of electronic health records, even among those implemented more than 15 years ago, highlighting: lack of accuracy and quality, complicated documentation process, competing interests and lack of functionalities^12^. On the other hand, another study that documented the nursing process in six German hospitals found as the main barriers: lack of motivation, insufficient technology for data collection at the bedside, low financial benefit at a high cost, failures or insufficient technology and lack of knowledge of (on) the programs^13^.

In the present study, item 20, which addresses naming and evaluation of the interventions and its accordance with the Nursing Interventions Classification (NIC), presented no significant improvement. This result demonstrates a particularity of the health institution itself, since it does not use the NIC taxonomy in its interventions. According to the guidelines of the Q-DIO instrument used in this study, item 20 should only receive the maximum score when the interventions present in the records are named according to the NIC^8^. It should also be noted that there is no computerized nursing process in the investigated institution.

The integration of the nursing process into the electronic health record has the potential to demonstrate the contribution of nursing to the health of individuals, raising the visibility of the profession and allowing the measurement of both the efficacy and the cost of nursing care^3^.

Regarding the concept Nursing Outcomes, only item 23 did not present significant improvement. However, it should be noted that the item already had a high value, and came close to being classified as fully documented. This item represents an ideal condition of care, and the evaluation/change of diagnoses daily or shift to shift is a fundamental part of the continuity of care.

A study that assessed nursing records and their implications on quality of care pointed out that the correct filling up of medical records from admission to discharge allows the nurse auditor, for example, to analyze the processes based on the hospital accreditation standards and document the indicators in which failures are found. Thus, it was possible to verify how the nursing records reflected the quality of the nursing care delivered to the patient and the continuity of the care provided by the nursing team^14^.

A study carried out in two hospitals with 843 nursing records evidenced that the records are deficient, do not portray the patient’s reality or the nursing care provided and do not contribute to the development of the nursing process. These data demonstrate that, in professional practice, nursing care is not always properly documented^15^. Also, when assessing continuity of care, the study found differences between the two hospitals surveyed^15^: in one, there was no logical sequence of information between one registry or another system which would allow the evaluation of the patient’s evolution by any health professional; in the other, 100% of the analyzed records presented continuity in the patient information, allowing the evaluation of the evolution of the clinical conditions^15^.

Thus, different studies have demonstrated weaknesses in the nursing documentation process^9^ ^,^ ^11^ ^,^ ^14^ ^-^ ^15^. However, other studies point to educational interventions allied to the use of taxonomies and electronic records as a way to improve the quality of nursing records ^(^ ^5^ ^-^ ^6^.

## Final considerations

The educational intervention proved to be effective at improving the quality of nursing records. Therefore, educational actions focused on real clinical cases may have positive implications for nursing practice.

It should be noted that, despite the effectiveness of the intervention, a lack of quality was identified in the evaluated records, revealed by the low mean values ​​of the items and by the weakness of some questions presented in the items, which did not present a significant increase. From this perspective, new studies focusing on the use of standardized language can be developed in the researched group. The use of electronic health record was suggested to the hospital and is in the process of implementation. Therefore, next studies should incorporate the three items - educational interventions, use of taxonomies and use of electronic records - in order to effectively improve the quality of nursing records, thus contributing to an effective, visible, quantifiable and qualified nursing care. 

## References

[1] Griffith R (2016). What to include in a nursing record. Br J Nurs.

[2] Marques DKA, Souza GLL, Silva AB, Silva AF, Nóbrea MML (2014). Conjunto Internacional de Dados Mínimos de Enfermagem: estudo
comparativo com instrumentos de uma clínica pediátrica. Rev Bras Enferm.

[3] Oliveira NB, Peres HHC, Jensen R, Yamasaki T (2014). Avaliação da qualidade dos registros eletrônicos de
enfermagem. PROENF: Gestão: Ciclo 4.

[4] Tastan S, Linch GFC, Keenan GM, Stifter J, McKinney D, Fahey L (2014). Evidence for the existing American Nurses Association-recognized
standardized nursing terminologies: A systematic review. Int J Nurs Stud.

[5] Müller-Staub M, Lunney M, Odenbreit M, Needham I, Lavin M, van Achterberg T (2009). Development of an instrument to measure the quality of documented
nursing diagnoses, interventions and outcomes: the Q-DIO. J Clin Nurs.

[6] Linch GFC, Müller-Staub M, Moraes MA, Azzolin K, Rabelo ER (2012). Cross-cultural adaptation of the quality of diagnoses, interventions
and outcomes (Q-DIO) instrument into Brazilian Portuguese. Int J Nurs Terminol Knowledge.

[7] Bruylands M, Paans W, Hediger H, Müller-Staub M (2013). Effects on the Quality of the Nursing Care Process Through an
Educational Program and the Use of Electronic Nursing
Documentation. Int J Nurs Terminol Knowledge.

[8] Odutayo PO, Olaogun AA, Oluwatosin AO, Ogunfowokan AA (2013). Impact of an Educational Program on the Use of Standardized Nursing
Languages for Nursing Documentation Among Public Health Nurses in
Nigeria. Int J Nurs Terminol Knowledge.

[9] Neves RS, Shimizu HE (2010). Análise da implementação da Sistematização da Assistência de
Enfermagem em uma unidade de reabilitação. Rev Bras Enferm.

[10] Rabelo-Silva ER, Dantas ACC, Ramos MCGC, Lucena AF, Almeida MA, Linch GFC (2017). Advanced Nursing Process Quality: Comparing International
Classification for Nursing Practice (ICNP) with the NANDA-International (NANDA-I)
and Nursing Interventions Classification (NIC). J Clin Nurs.

[11] Barral LNM, Ramos LH, Vieira MA, Dias OV, Souza LPS (2012). Análise dos Registros de Enfermagem em prontuários de pacientes de um
hospital de ensino. Rev Min Enferm.

[12] Vabo G, Slettebb A, Fossum M (2016). Nursing Documentation: An Evaluation of an Action Research
Project. Stud Health Technol Inform.

[13] Vollmer AM, Prokoscha HU, Bürklea T (2014). Identifying Barriers for Implementation of Computer Based Nursing
Documentation. Stud Health Technol Inform.

[14] Morais CGX, Batista EMS, Castro JFL, Assunção SS, Castro GMO (2015). Registros de enfermagem em prontuário e suas implicações na qualidade
assistencial segundo os padrões de acreditação hospitalar: um novo olhar da
auditoria. Rev ACRED.

[15] Silva TG, Santos RM, Crispim LMC, Almeida LMWS (2016). Conteúdo dos registros de enfermagem em hospitais: contribuições para
o desenvolvimento do processo de enfermagem. Enferm Foco.

